# SLC25A22 promotes proliferation and metastasis by activating MAPK/ERK pathway in gallbladder cancer

**DOI:** 10.1186/s12935-019-0746-9

**Published:** 2019-02-14

**Authors:** Pengcheng Du, Haibin Liang, Xiaowei Fu, Peng Wu, Chao Wang, Haimin Chen, Bingbing Zheng, Jun Zhang, Shuanghui Hu, Rengui Zeng, Bo Liang, Lu Fang

**Affiliations:** 1 Department of General Surgery, Second Affiliated Hospital of Nanchang University, No. 1 Minde Road, Nanchang, 330006 China; 2 Jiangxi Province Key Laboratory of Molecular Medicine, No. 1 Minde Road, Nanchang, 330006 China; 3 Department of General Surgery, Xinhua Hospital Affiliated to Shanghai Jiao Tong University School of Medicine, No. 1665 Kongjiang Road, Shanghai, 200092 China; 4 Department of General Surgery, First Affiliated Hospital of Nanchang University, No. 17 Yongwai Main Street, Nanchang, 330006 China

**Keywords:** Gallbladder cancer, SLC25A22, Mitochondrial apoptosis, MAPK/ERK

## Abstract

**Background:**

SLC25A22, a member of mitochondrial carrier system (MCS) family encoding a mitochondrial glutamate transporter, has been reported to have vital roles in promoting proliferation and migration in cancer. Gallbladder cancer (GBC) is the most common biliary tract malignancy and has a poor prognosis. We aimed to determine the expression and function of SLC25A22 in GBC.

**Methods:**

Immunohistochemistry (IHC) staining analysis and quantitative real-time PCR (qRT-PCR) were conducted to determine the expression of SLC25A22 in GBC tissues. Human NOZ and GBC-SD cells were used to perform the experiments. The protein expression was detected by western-blot analysis. Cell viability was evaluated via CCK-8 assay and colony formation assay. Cell migration and invasion in vitro were investigated by wound healing and transwell assay. Annexin V/PI staining assay for apoptosis were measured by flow cytometry. The effect of SLC25A22 in vivo was conducted with subcutaneous xenograft.

**Results:**

We indicated that the expression of SLC25A22 was significantly upregulated in GBC tumor tissues as well as cell lines. Downregulation of SLC25A22 inhibited GBC cell growth and proliferation in vitro and in vivo and also had an effect on metastasis of GBC cells through the EMT processes. In addition, inhibition of SLC25A22 promoted mitochondrial apoptosis via downregulating BCL-2 and upregulating cleaved PARP, Cytochrome-c, and BAX mediated by MAPK/ERK pathway.

**Conclusions:**

Our study identified that SLC25A22 promoted development of GBC activating MAPK/ERK pathway. SLC25A22 has a potential to be used as a target for cancer diagnosis of GBC and related therapies.

## Background

Gallbladder cancer (GBC) is the most common biliary tract malignancy and the fifth most common gastrointestinal cancer [1]. The poor prognosis of GBC is due to its usually delayed diagnosis and early metastasis, as the 5-year survival rate is only ~ 5% [2, 3]. Therefore, there a limited time period in which a patient can receive surgical treatment. As a result, a better understanding of the molecular mechanism of GBC is indispensable.

The 53-member canonical SLC25A transporter groups and several identified noncanonical transporters are involved in the mitochondrial carrier system (MCS) in mammals. This system transports small molecules between the mitochondria and cytoplasm [4]. SLC25A22 specifically, as a member of MCS family, was identified to encoded a mitochondrial glutamate transporter [5]. Recent studies have reported on the potential effects of SLC25A22 in neonatal epileptic encephalopathy and migrating partial seizures in infancy [6–9]. In addition, Wong and Li et al. revealed that the overexpression of SLC25A22 in colorectal cancer (CRC) and its vital roles in promoting proliferation and migration of CRC with mutation KRAS. Meanwhile, elevated expression of SLC25A22 was associated with a poor prognosis [10]. However, there is little research about SLC25A22 in malignant tumors and no study of effects and mechanism of SLC25A22 in GBC.

In this study, we determined the expression of SLC25A22 in GBC and further explored its function in the GBC cell. Moreover, we suggested that MAPK/ERK pathway was affected by SLC25A22 thereby mediating mitochondrial apoptosis to promote malignant behaviors in GBC. Taking together, SLC25A22 has potential to be a prognostic and therapeutic biomarker in GBC.

## Methods

### Cell culture

The GBC lines used in this study were as follows: GBC-SD, SGC-996 were purchased from Shanghai Institute for Biological Science, Chinese Academy of Science (Shanghai, China). NOZ, OCUG-1, EHGB-1, EHGB-2 were purchased from the Health Science Research Resources Bank (Osaka, Japan). GBC-SD, EHGB-1, EHGB-2, OCUG-1 cells were cultured separately in DMEM (Gibco) with 10% fetal bovine serum (FBS, Gibco) and 1% penicillin–streptomycin (Gibco). NOZ cells were cultured in William’s medium E (Lonza) with 10% fetal bovine serum (FBS, Gibco) and 1% penicillin–streptomycin (Gibco). Contrastingly, SGC-996 cells were cultured in RPMI 1640 medium (Hyclone) with 10% fetal bovine serum (FBS, Gibco) and 1% penicillin–streptomycin (Gibco). All of above cells were cultured in their respective media in a humidified incubator at 37 °C with 5% CO_2_.

### Immunohistochemistry (IHC)

Immunohistochemical staining of GBC and paracancerous tissue was performed using anti-SLC25A22 (Abcam, 1:200), and subcutaneous xenograft of mice using anti-Ki67 (Abcam, 1:200) and anti-p-ERK (CST, 1:200). The sections were incubated with primary antibody overnight at 4 °C after deparaffinization and antigen recovery. Then, the sections were incubated with a secondary antibody (Beyotime, 1:2000) for about 40 min at 20 °C. Sections were scored as previously described [11].

### Quantitative real-time PCR (qRT-PCR)

Total RNA was extracted from tissues by using Trizol reagent (Invitrogen), thus, the cDNA products were synthesized using PrimeScript Reverse Transcriptase (Takara) according to the manufacturer’s protocol. All products were amplified by PCR and measured using SYBR^®^ Green (Takara), the expression results were analyzed by StepOnePlus™ Real-time PCR system (Applied Biosystems). The primer sequences for SLC25A22 were shown as follows: 5′-primer, GGGAGTTCACCGGACATCTG and 3′-primer, CCCATTCAATCTCCCGCTGT. The relative expression was estimated with 2^−ΔΔCT^ method, using GAPDH as an internal control.

### Migration and invasion assays

Cell migration and invasion were assessed using transwell migration and invasion chambers (BD Biosciences). Cells were transfected with shRNA-SLC25A22 and shRNA-NC were resuspended in serum-free medium and then seeded respectively into the upper chambers, while the lower chambers was maintained in 10% FBS medium. After 20 h of incubation, cells on the bottom of chambers were fixed with 4% paraformaldehyde for 30 min and dyed with crystal violet for 15 min. The images of stained cells were captured using a microscope (Leica).

### Cell viability assay

Cell viability was assessed using Cell Counting Kit-8 (CCK-8, Yensen) according to the manufacturer’s instructions. Firstly, cells were separately seeded into 96-well plates at a density of 1000 per well. After incubation with CCK-8 for 2 h, the absorbance of cells was measured at a wavelength of 450 nm by a microplate reader (BioRad), all of above were conducted at various times (6 h, 24 h, 48 h, 72 h, 96 h). The results were demonstrated in the form of a line chart.

### Colony formation and wound-healing assay

Cells were separately plated into six-well plates at a density of 1000 per well with 10 days of incubation to conduct colony formation. Cell formations were fixed with 4% paraformaldehyde for 30 min, and subsequently stained with crystal violet and the total number of colonies (more than 50 per colony) was counted. For wound-healing assay, cells were seeded into six-well plates and grown to confluence, then wounds were created by scratching confluent cell monolayers with a 200-μl pipette tip and incubating them with 10% FBS medium in incubator at 37 °C with 5% CO_2_. Images were taken at 0 h and 24 h under a microscope (Leica). The percentage (%) change in migration was determined via comparison of the difference in wound width.

### Western-blot

Total protein was extracted from cells using RIPA buffer (Beyotime) at 4 °C about 20 min while the PMSF (Beyotime) was used for the inhibition of protein degradation. Equal amounts of sample protein (15–20 µg per lane) were separated by SDS-PAGE and transferred onto 0.45 µm PVDF membranes (Millipore). After blocked with 5% milk at 20 °C about 1 h, membranes were incubated with the corresponding primary antibody at 4 °C overnight, followed by three times wash in TBST for 10 min. After that, the membranes were incubated with HRP-conjugated secondary antibodies (Beyotime) at 20 °C. Protein bands were exposed by the Gel Doc 2000 (BioRad). The primary antibodies mentioned above were for: SLC25A22 (Abcam, 1:1000), E-cadherin (Abways, 1:1000), N-cadherin (Abways, 1:1000), vimentin (CST, 1:1000), ERK (CST, 1:1000), p-ERK (CST, 1:1000), MEK (CST, 1:1000), p-MEK (CST, 1:1000), GAPDH (Abways, 1:1000), BCL-2 (ABclonal, 1:1000), cytochrome-c (CST, 1:1000), PARP (CST, 1:1000), cleaved PARP (CST, 1:1000).

### Lentivirus and cell infection

For the knockdown of SLC25A22, a short hairpin RNA (shRNA-SLC25A22) sequence complementary to SLC25A22 (5′-GTGGTGTACTTCCCGCTCTTT-3′) and a scramble sequence were synthesized by Genomeditech. Cells were harvested 48 h after transfection of lentivirus. When green fluorescence (> 80%) was observed under fluorescence microscope, which indicated the establishment of stable transfection. The efficiency of this shRNA was examined by western-blot as mentioned previously.

### Subcutaneous xenograft

Fourteen BALB/c nude mice (average 4 weeks-old and 20 g) were purchased from the Shanghai Laboratory Animal Center of the Chinese Academy of Sciences (Shanghai, China) and were divided into two groups randomly. NOZ cells (Lv-NC/Lv-SLC25A22) were injected into the armpit of each mouse at a density of 1 × 10^6^/100 µl. Tumor volumes were measured twice a week from what they were visible. About 4 weeks later, all mice were killed and tumor tissues were fixed by 4% paraformaldehyde for IHC staining analysis. All operations as described above were approved the Institutional Animal Care and Use Committee of Xinhua Hospital, School of Medicine, Shanghai Jiao Tong University.

### Cell apoptosis assay

An Annexin V/PI Apoptosis Kit (BD Biosciences) was used for cellular apoptosis analysis, NOZ cells (Lv-NC/Lv-SLC25A22) were harvested and washed with cold phosphate buffer saline (PBS), then the cells were resuspended in 1 × Binding Buffer at a concentration of 1 × 10^6^ cells/ml and 100ul of the solution (1 × 10^5^ cells) was transferred to a 5 ml culture tube. After incubation with 5 μl FITC Annexin V and 5 μl PI in the dark for 15 min at 25 °C, the samples were mixed into 400 μl 1 × Binding Buffer and immediately analyzed by flow cytometry (BD Biosciences).

## Results

### SLC25A22 overexpression be related with poor prognosis in GBC

**Fig. 1:**
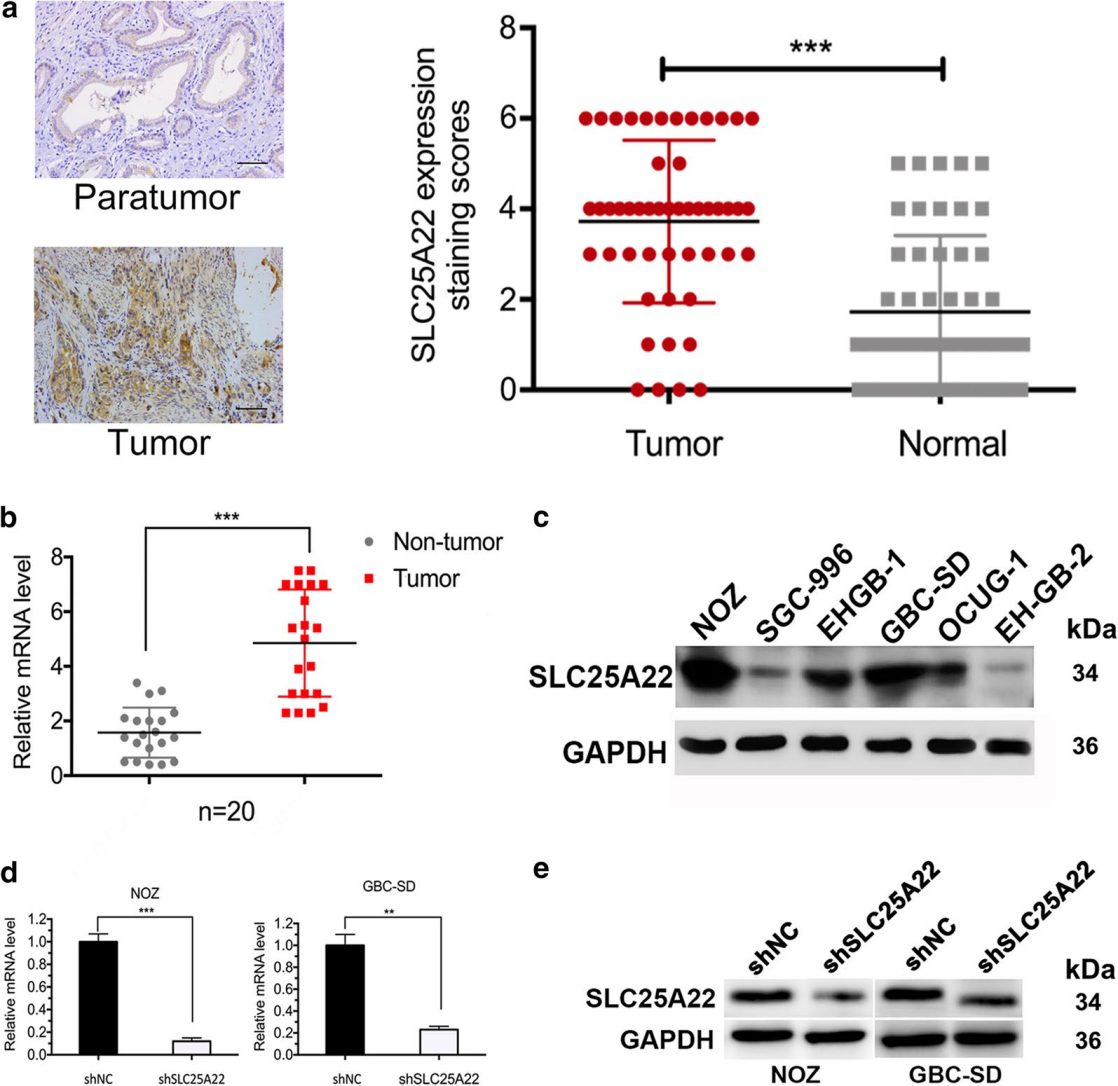
SLC25A22 is overexpressed in gallbladder cancer (GBC). **a** Immunohistochemical staining of tumor tissues and corresponding adjacent normal tissues for SLC25A22. Expression staining scores are shown in the graph bar (*n *= 40) {*p *< 0.001}. **b** Levels of relative SLC25A22 mRNA were detected in 20 GBC tumor tissues and corresponding normal tissues by qRT-PCR (*n *= 20) {*p *< 0.001}. **c** NOZ and GBC-SD display higher expression of SLC25A22 than others, as observed by western-blot. **d**, **e** Knockdown efficiency of an shRNA specifically directed against SLC25A22 was tested in protein and mRNA level. An shRNA blocked relative mRNA of SLC25A22 compared to control (shNC) in NOZ and GBC-SD using qRT-PCR, western-blot. The expression of SLC25A22 protein was significantly decreased in NOZ and GBC-SD transfected with shRNA. GAPDH was used an internal reference

Research of Wong et al. showed that SLC25A22 had a critical role in promoting proliferation and migration in colorectal cancer. In the light of our microarray results, we hypothesized that overexpression of SLC25A22 may play a similar role in GBC. To test this conjecture we compared the expression of SLC25A22 in GBC tissues and corresponding adjacent normal tissues via IHC (Fig. 1a). We found that the expression of SLC25A22 in tumor tissues was significantly higher than that in adjacent tissues. In addition, the results of PCR also demonstrated that there was a difference at the RNA level (Fig. 1b).

### SLC25A22 expression level in GBC cell lines

Western-blot assay was used to detect the protein expression level in six gallbladder cancer cell lines (NOZ, GBC-SD, EHGB-1, SGC-996, OCUG-1, EHGB-2). As a result, GBC-SD and NOZ were chosen as candidates because they had higher level of SLC25A22 than other cell-types (Fig. 1c). To validate the function of SLC25A22 in GBC cells, we synthesized a lentiviral shRNA specially against SLC25A22 which could effectively suppress the expression of SLC25A22. Knockdown efficiency of SLC25A22 were tested in mRNA and protein level by qRT-PCR and western-blot (Fig. 1d, e).

### Knockdown of SLC25A22 inhibited GBC cell growth and proliferation in vitro and in vivo

**Fig. 2:**
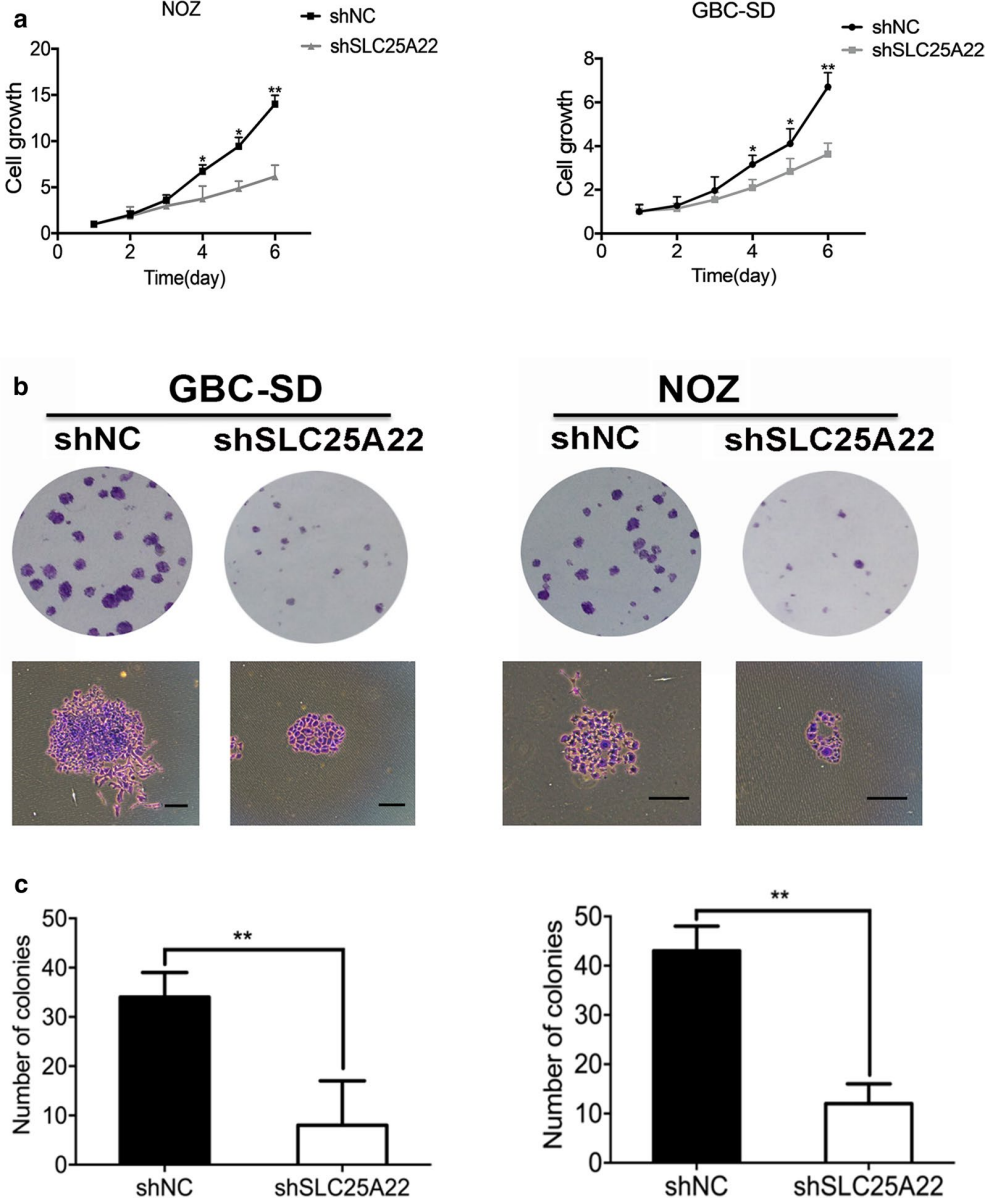
Block of SLC25A22 inhibited growth in vivo. **a** The viability of GBC-SD and NOZ transfected with shSLC25A22 and shNC were analyzed using CCK8 viability assays (*p < 0.05, **p < 0.01). **b**, **c** Cell colony formation displayed cell growth suppression when SLC25A22 was knocked down (shSLC25A22) compared to the control group (shNC). The number of colonies are shown in charts (**p < 0.01)

In order to reveal the role of SLC25A22 in cell growth and proliferation, we downregulated the expression of SLC25A22 in GBC-SD cells and NOZ cells via transfecting with lentiviral shRNA. Cell viability was tested by CCK-8 assay and colony formation assay was conducted as described previously (Fig. 2). In contrast, downregulation of SLC25A22 obviously inhibited the viability of GBC-SD cells and NOZ cells. Moreover, colony formation assay demonstrated that the number of colony form was also decreased by the block of SLC25A22.

**Fig. 3:**
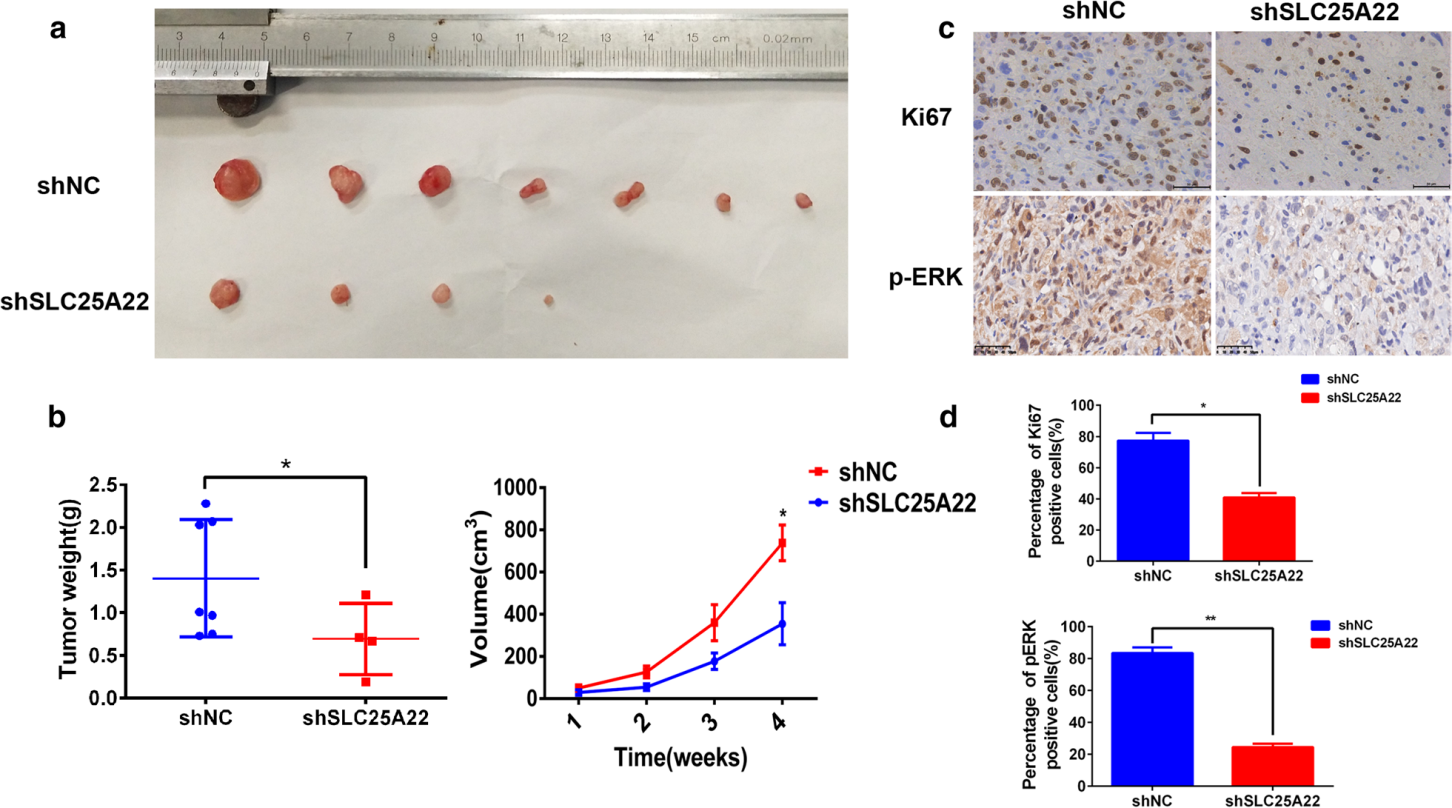
SLC25A22 plays a vital role in GBC metastasis and proliferation in vivo. **a** SLC25A22 inhibited cell growth in vivo as observed with a subcutaneous xenograft model. **b** The tumor volumes were measured once a week from the 10th day and the results are shown on the line chart (*p < 0.05, **p < 0.01). Tumor were weighed when the mice were sacrificed and the data was displayed on the scatter plot chart (*p < 0.05). **c**, **d** IHC staining of tumor xenografts for Ki67 and p-ERK. The percentage of Ki67 and p-ERK positive cells are shown in the graph bar (*p < 0.05, **p < 0.01)

In order to further verify the suppressive effect on tumor growth in vivo via downregulation of SLC25A22, we established a stable NOZ cell line with low expression of SLC25A22 by transfecting lentiviral shRNA while shNC was a control. Then the two groups of NOZ cells (shSLC25A22/shNC) were subcutaneously injected into node mice and the rate of tumor growth were recorded (Fig. 3a, b). As expected, knockdown of SLC25A22 significantly inhibited the subcutaneous xenograft growth. Consistently, IHC staining of the proliferative index Ki67 and p-ERK displayed a similar result (Fig. 3c, d). In summary, SLC25A22 may play a crucial role in GBC cell growth and proliferation.

### Downregulation of SLC25A22 suppressed metastasis of GBC in vitro via EMT

**Fig. 4:**
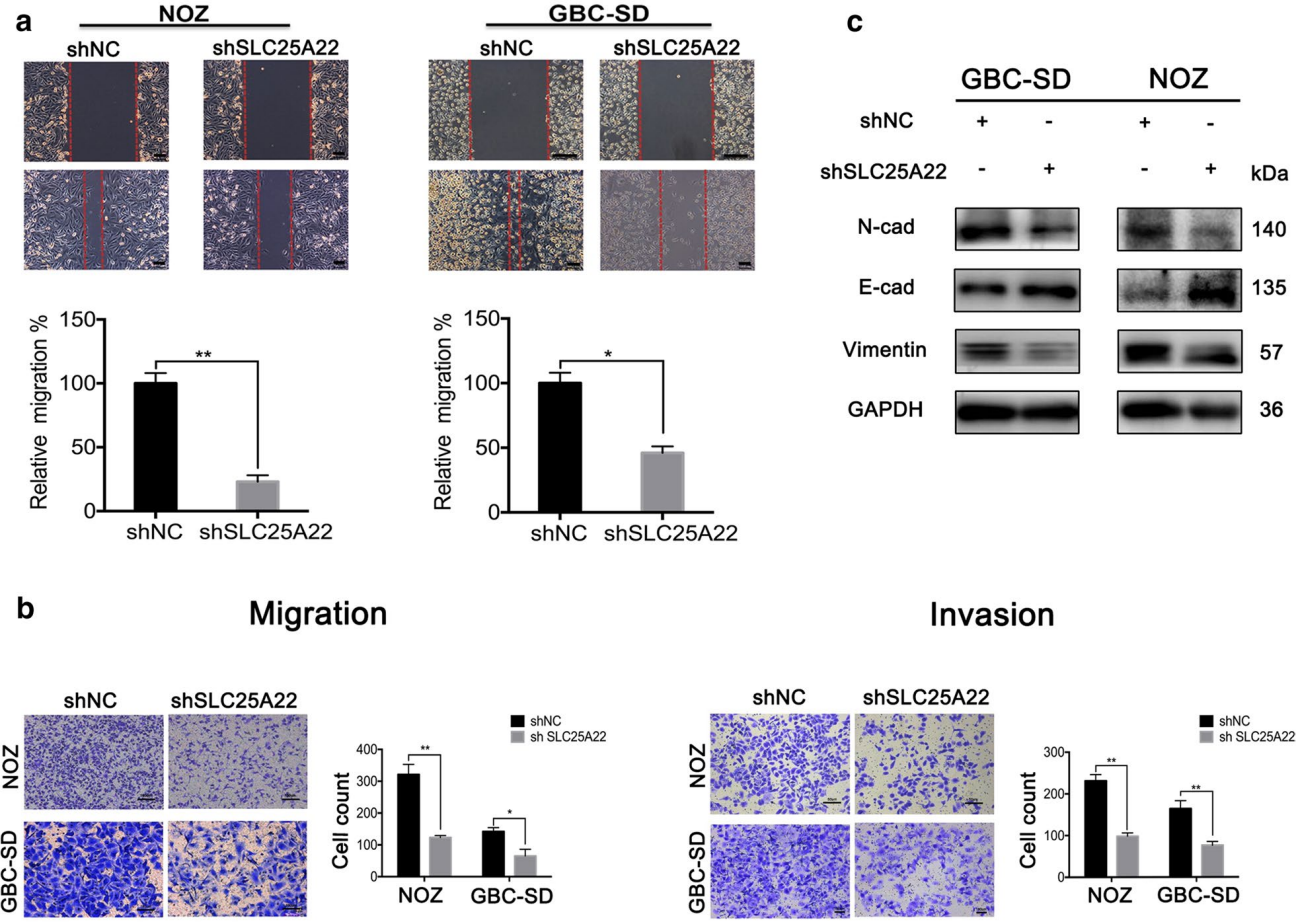
SLC25A22 promotes cell migration and invasion by inducing EMT in GBC. **a** Representative results of wound-healing for GBC-SD and NOZ cells transfected with shNC and shSLC25A22. Relative migration are shown in relevant graph bars (*p < 0.05, **p < 0.01). **b** The typical pictures of migration and invasion transwell assay for GBC-SD and NOZ cells transfected with shNC and shSLC25A22 are displayed. Cell counts are shown in relevant graph bars (*p < 0.05, **p < 0.01). **c** EMT-related proteins (E-cadherin, N-cadherin, vimentin) expression were evaluated by western-blot, and GAPDH was used as an internal reference

To validate whether the metastasis was affected by SLC25A22 in GBC cells, a wound healing assay and transwell assay were conducted in GBC-SD and NOZ cells transfected with shSLC25A22/shNC. As shown in Fig. 4a, b, migration and invasion ability of the shRNA group was dramatically suppressed compared with control group. Furthermore, we examined the expression of EMT-related protein (E-cadherin, N-cadherin, Vimentin) in groups above mentioned using western-blot (Fig. 4c), which suggests that SLC25A22 may have an effect on metastasis of GBC cells through the EMT processes.

### Inhibition of SLC25A22 promoted mitochondrial-related apoptosis in GBC cells via MAPK/ERK pathway

**Fig. 5:**
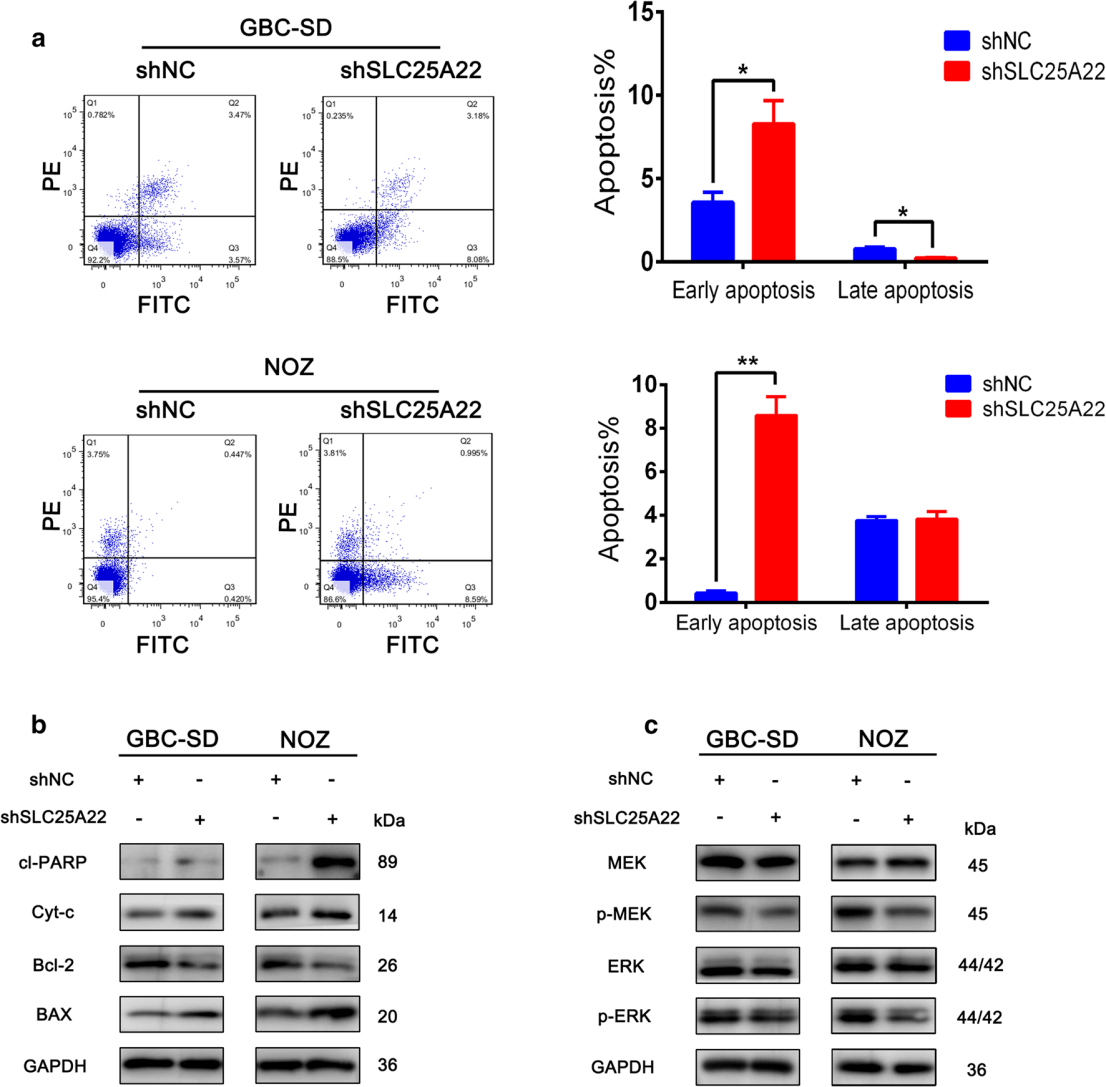
SLC25A22 inhibits GBC cells apoptosis by regulating MAPK/ERK pathway. **a** Flow cytometry analysis of cell apoptosis of GBC-SD and NOZ cells with annexin V/PI staining indicating there was a significant difference of early apoptosis between two groups (shNC, shSLC25A22). **b** Apoptosis-related proteins (cleaved-PARP, Cytochrome-c, BCL-2, BAX) expression was analyzed by western-blot. **c** The protein of GBC-SD and NOZ cells were transfected with shNC and shSLC25A22 was subjected to analysis by western-blot. Representative immunoblots of MAPK/ERK pathway were analyzed with GAPDH as an internal reference

In light of the suppressive effect on GBC cell progression by downregulating SLC25A22, we separately carried out an apoptosis analysis for two groups of GBC-SD and NOZ cells (shSLC25A22/shNC) using Annexin V/PI Apoptosis Kit and flow cytometry (Fig. 5a). Our results indicated that downregulation of SLC25A22 led to a significant increase in the number of early apoptotics cells. To further explore the molecular mechanism for this alteration, the expression of several apoptosis markers were detected by western-blot (Fig. 5b). Cleaved-PARP, Cytochrome-c and BAX, three markers representing a state of apoptosis, were found to be significantly increased in the shRNA group. In contrast, BCL-2 was apparently reduced by knockdown of SLC25A22. It has been reported that the deregulation of MEK/ERK phosphorylation plays an antiapoptotic role in various cancers [12–17]. To determine whether the MAPK/ERK signaling pathway is associated with SLC25A22-induced apoptosis, we estimated the protein levels of MEK/ERK using western-blot and indicated that the expression of p-MEK/p-ERK was obviously decreased when SLC25A22 was downregulated (Fig. 5c). Therefore, MAPK/ERK pathway was considered to play a vital role in apoptosis by knockdown of SLC25A22.

## Discussion

Our results demonstrated there was high expression of SLC25A22 in gallbladder cancer and that it played a key role it played tumor progression including in vitro and in vivo. We focused on SLC25A22, a gene encodes mitochondrial glutamate transporter, that was shown by Wong et al. to promote proliferation and migration in colorectal cancer [10]. However, most research about SLC25A22 concentrated on its association neonatal epileptic encephalopathy [6–9], so the function of SLC25A22 and its molecular mechanism still remain unclear in malignant tumors. Interestingly, our microarray analysis demonstrated that there was an expression difference of SLC25A22 in GBC. The result of IHC staining and qRT-PCR analysis confirmed that SLC25A22 was a tumor-related protein.

As is well known, the aggressive proliferation capacity of metastasis is the most devastating feature of malignant tumor. Gallbladder cancer is a highly aggressive fatal neoplasm characterized by its strong invasiveness and rapid progression [18]. In the present study, the result of our experiments indicated that the metastasis and proliferation of the tumor were effectively inhibited by downregulating the expression of SLC25A22. The epithelial-to-mesenchymal transition (EMT) is a process related with tumor stemness, metastasis and resistance to therapy [19]. In our experiments, SLC25A22 was shown to be a significant factor in promoting tumor invasion and migration. E-cadherin, N-cadherin and vimentin, are prominent markers of EMT [20]. Interestingly, our western-blot analysis demonstrated there was a close relationship between SLC25A22 and EMT. In other words, SLC25A22 might promote tumor cell invasion and metastasis via the EMT pathway in GBC.

The microenvironment of cancer cells and surrounding stromal cells with different genetic/epigenetic backgrounds is distinct from normal cells, which is called “intra-tumoral heterogeneity” [21]. The Warburg effect described as a tendency of most cancer cells to uptake glucose and convert it primarily to lactate in spite of available oxygen in cancer cells. Even though the Warburg effect has been widely recognized as an important feature of metabolic reprogramming, a lot of evidence has displayed that cancer cells also depend on mitochondrial metabolism [22, 23]. It has been reported that the oncogene c-Myc, which is known to engage in glutamine catabolism that exceeds requirement for protein and nucleotide biosynthesis by its transcriptional regulatory roles of repressing miR-23a and miR-23b [24]. As a result of c-Myc-mediated metabolic reprogramming, both glutamine uptake and glutamine catabolism are increased therefore the TCA cycle in mitochondria are frequently activated, which is called “glutamine addiction” [21, 25]. This is why the elevated expression and interaction of amino acid transporters, contributes to the activation of glutamine metabolic reprogramming and protects tumor cells against accumulation of oxidative stress mediated by cystine metabolic reprogramming. However, the complex mechanism of metabolic reprogramming due to c-Myc expression that regulates glutamine metabolism in cancer cells is still not well understood. Programmed cell death, also called apoptosis, was first defined by Lockshin & Williams in the insect development [26]. Numerous studies have supported the conclusion that apoptosis plays a key role in regulating the development of malignant tumors. Mitochondrial outer membrane permeabilization (MMOP), a crucial process in intrinsic apoptotic pathway, was mediated by BCL-2-associated X protein (BAX) [27]. As a result, cytochrome-c was released from mitochondrial intermembrane to cytoplasm and induced to form apoptosome which indicated the occurrence of apoptosis [28]. In addition, cleaved PARP (poly-ADP-ribose polymerase) was highly considered to be a marker of activation in apoptosis [29, 30]. Yet the MMOP process was inhibited by the anti-apoptotic member BCL-2. Due to the results of cell apoptosis analysis, SLC25A22 may promote tumor growth by suppressing the mitochondrial apoptosis in GBC. To further validate our opinion, we detected the proteins related with intrinsic apoptotic pathway as mentioned above by western-blot. Consistently, SLC25A22 promoted tumor progression by preventing the process of apoptosis via the mitochondrial pathway.

According to research by Wong et al., SLC25A22 knockdown caused the depletion of ATP as it activated AMP-activated protein kinase α (AMPKα) so that RAF/MEK/ERK cascade was suppressed in CRC. Therefore, we focused on the MAPK/ERK pathway, a classical signaling pathway reported to regulate tumor development including proliferation, EMT, apoptosis, inflammation, and immunity [31–36]. The previous study has confirmed that MAPK/ERK was involved in BAX-mediated apoptosis. In the present study, we further demonstrated that downregulation of SLC25A22 significantly suppressed p-MEK/p-ERK protein expression. Combined with the role of SLC25A22 in mitochondria-mediated apoptosis, activation of MAPK/ERK pathway directly led to a delay in apoptosis in GBC thereby contributing to its malignant characteristics.

## Conclusions

In conclusion, our study identified that overexpression of SLC25A22 was related with poor prognosis in GBC. Moreover, SLC25A22 promotes development of GBC via altering mitochondria-associated apoptosis process mediated by MAPK/ERK pathway. Thus, SLC25A22 has a potential to be used as a target for cancer diagnosis of GBC and related therapies.

## Abbreviations

MCS: mitochondrial carrier system
GBC: gallbladder cancer
qRT-PCR: quantitative real-time PCR
IHC: immunohistochemistry
MAPK: mitogen-activated protein kinase
ERK: extracellular regulated protein kinases
pERK: phosphorylated extracellular regulated protein kinases
CRC: colorectal cancer
FBS: fetal bovine serum
CCK-8: cell counting kit-8
HRP: horse radish peroxidase
PRAP: poly ADP-ribose polymerase
BCL-2: B-cell lymphoma-2
BAX: B-cell lymphoma-2-associated X protein
GAPDH: glyceraldehyde-3-phosphate dehydrogenase
FITC: fluorescein isothiocyanate
PI: propidium iodide
PBS: phosphate buffer saline
EMT: epithelial-to-mesenchymal transition
MMOP: mitochondrial outer membrane permeabilization
AMPKα: AMP-activated protein kinase α
